# Myofunctional device use in oral care and swallowing: a protocol for a feasibility study in an aged care population

**DOI:** 10.1186/s40814-022-01148-3

**Published:** 2022-08-19

**Authors:** Hollie-Ann L. Shortland, Sally Hewat, Gwendalyn Webb, Anne E. Vertigan

**Affiliations:** 1 Speech Pathology, School of Health Sciences, College of Health, Medicine & Wellbeing, University of Newcastle, Newcastle, Australia; 2 John Hunter Hospital, Newcastle, Australia

**Keywords:** Speech-language pathology treatment, Myofunctional device, Oral hygiene, Dysphagia

## Abstract

**Background:**

Poor oral health is a known predictor of aspiration pneumonia in vulnerable populations such as the elderly and chronically ill and has been linked to systemic disease, morbidity, and mortality. Reduced oral health not only places individuals at a greater risk of aspiration pneumonia but may result in pain or poorer dentition which can impact on mastication and swallowing. Consequences of this may include reduced oral intake, malnutrition, poorer health outcomes, and reduced quality of life. Few evidence-based protocols exist to manage oral care in aged care populations, and maintenance of good oral hygiene is difficult for nursing and care staff to facilitate. However, a recent literature review found that improvements in oral hygiene, oral behaviors, and swallowing, along with breathing and speech have been found to be associated with the use of myofunctional devices due to positive changes in orofacial functions such as lip seal, mastication, swallowing, and nasal breathing patterns. The primary aim of this study is to assess the feasibility of using a myofunctional device to improve oral care and swallowing function in an aged care population.

**Methods/design:**

This project is a feasibility study that involves a 5-week intervention for oral hygiene and dysphagia for residents >65 years old in an aged care setting. Feasibility will be determined by the acceptability of the intervention, study recruitment and retention, and adherence to the intervention. Feasibility testing will also include an evaluation of clinical outcome measures, and sensitivity to detect changes in oral health and swallowing in an aged care population.

**Discussion:**

The results of this trial will provide important information regarding the feasibility of utilizing a myofunctional device to improve oral care and dysphagia in elderly patients in an aged care facility. This knowledge will further guide and inform design of a larger trial or future research.

**Trial registration:**

This trial was registered August 10, 2021, with the Australian New Zealand Clinical Trials Registry and allocated the ACTRN: ACTRN12621001359820.

**Supplementary Information:**

The online version contains supplementary material available at 10.1186/s40814-022-01148-3.

## Background

The link between poor oral health with systemic disease, morbidity, and mortality has been demonstrated extensively throughout allied health, nursing, and dental literature [1–4]. Oral health is important in both children and adults. Poor oral health, such as tooth decay, gum disease, and tooth loss, contributed to 4.5% of non-fatal diseases in Australia in 2015, with 78% of oral disorders in people aged 85 years and over relating to poor oral health [5]. Oral health deteriorates over a person’s lifetime, with children aged 5–10 years of age on average having 1.5 decayed, missing or filled teeth. In contrast, an adult 75+ years on average has 24.4 decayed, missing, or filled teeth [6]. The rate of tooth decay has been reported to be higher in the indigenous population and those in rural and remote areas in Australia [7]. Early-stage gum disease, known as gingivitis, is caused by an accumulation of plaque on the teeth and gum line. It can cause inflammation and irritation to the gums which if left untreated leads to more serious periodontal disease, including damage to the soft tissue and loss of teeth [8]. In 2017–2018, the proportion of adults with advanced stage gum disease, periodontitis, increased with age from 51% in those 55–74 years, to 69% in those 75 years and over [5]. Further to this, the Australian Institute of Health and Welfare (AIHW) [6] reported that between 2017 and 2018, there were approximately 72,000 hospitalizations in Australia for dental conditions that may have been prevented with earlier treatment.

Factors that influence poor oral health are consumption of sugar, alcohol, and tobacco; reduced access to dental services; and reduction in good oral hygiene [6]. The Australian Health Ministers Advisory Council (AHMAC) [7] identified four population groups that are at greater risk of poor oral health, including socially disadvantaged or low income; Aboriginal and Torres Strait Islander Australians; those living in rural and remote areas; and people with additional and or specialized health care needs such as those with mental illness or the frail older population. Reduced oral health and an inability to manage oral heath independently is a predictor of aspiration pneumonia in vulnerable populations such as the elderly and chronically ill [9]. If oral health is reduced, not only are people at greater risk of aspiration pneumonia, but they may also have pain or poorer dentition. This impacts on mastication and swallowing, with potential for malnutrition, poorer health outcomes, and reduced quality of life [10]. Reduced oral health is associated with a number of chronic diseases including stroke and cardiovascular disease [11].

There are several factors that impact on oral hygiene and swallowing ability. An oral breathing pattern may result in a dry oral cavity and reduced oral hygiene, which further impacts on swallowing and may increase the risk of aspiration pneumonia [12]. Reduced oral hygiene may lead to pain and/or increased difficulties with mastication, further leading to decreased muscle bulk and poor tolerance of diet consistency [12]. The need for change of diet consistency may be influenced by the risk of aspiration or choking, and in populations such as the elderly, these diet changes may lead to an increased risk of malnutrition, dehydration, and consequently, a reduction in quality of life [13]. Researchers [12] explored the impact of myofunctional devices on speech, swallowing, and quality of life in the elderly, noting that a reduction in lip tone in the elderly population may further influence an oral breathing pattern over nasal breathing, which can result in a dryer oral cavity. The lack of knowledge of oral healthcare in nursing staff in Australia was noted by Ajwani and colleagues [3] in their scoping review of integrated oral care for stroke patients. This scoping review of the literature highlighted the importance of oral health post-stroke in reducing the risk of aspiration pneumonia, the need for integrated oral health programs, and discussed the need for maintaining optimal oral health due to the links between gingivitis and cerebrovascular infarction as well as periodontal disease and stroke [3].

Currently, there are limited oral care protocols with measurable outcomes that are used in hospitals and care facilities in Australia [14]. These protocols have been described as ad hoc and often not prioritized in patient care [14]. This is of concern to speech pathologists due to the impact of poor oral health on mastication, swallowing function, and increased risk of aspiration. However, a recent literature review highlights improvement in swallowing function, oral behaviours, speech, and oral hygiene with the use of orofacial myofunctional therapy and myofunctional devices [15, 16]. The positive changes in orofacial functions such as lip seal, mastication, swallowing, and nasal breathing patterns are important in maintaining good oral hygiene which is identified as being problematic in an aged care population [12]. A review by Slack-Smith and colleagues [17] in aging and oral health indicated that on entering aged care facilities many older people are in urgent need of oral care. However, there continues to be barriers to improving oral health due to reduced health literacy, lack of knowledge and understanding, cost, and complex medical conditions [7, 17].

In a study by Shortland and colleagues (manuscript in preparation) into speech pathologists’ use and outcomes of myofunctional devices in therapy programs, there was found to be both similarities and differences in the use of myofunctional devices, and the therapy programs in which these devices are incorporated. This varied across intervention areas, caseloads, and diagnoses amongst speech pathologists who utilized them. Shortland and Colleagues (manuscript in preparation) noted that the type of myofunctional device, timing of introduction, utilization in isolation, adherence, and dosage variation of myofunctional devices used contributed to successful outcomes for swallowing, oral hygiene, breathing, and speech. However, further education and research into myofunctional device use and guidelines to direct their use in speech pathology intervention was recommended, along with a coordinated approach and team input in assessment and intervention.

Despite this increase of evidence, there is limited research in speech pathology that addresses the use and outcomes of myofunctional devices in clinical practice [15]. The potential impact of improvement on orofacial function, including oral hygiene and swallowing, has already been identified with the use of myofunctional devices in literature from various health disciplines [18]. As well as this, there is a link between reduced oral hygiene and aspiration pneumonia [19] and the impact of a reduction in oral hygiene on quality of life [20]. It is relevant to further explore treatment dosage, utilization, and outcomes of a myofunctional device in a population such as the elderly who make up a large proportion of those whose oral health and swallowing function may be impacted on [21].

Given the rapid rise in an aging population [22], the limited access to oral health systems in an aged care facilities [23], and increased dependency on care once entering a facility [24], it would be appropriate to consider the use of myofunctional devices in oral health and swallowing due to the reported improvement in orofacial function [18]. This study will look at the feasibility of using myofunctional devices in oral health and swallowing for an aged care population to inform a larger study.

## Method

### Design overview

This is a single-arm study, designed to examine the feasibility of the use of a myofunctional device in improving in oral health and dysphagia in a residential, aged care population. The protocol presented is based on both the Standard Protocol Items: Recommendations for Interventional Trials (SPIRIT) [25] and Consolidated Standards of Reporting Trials (CONSORT) [26] guidelines, to conduct a feasibility study (Additional file 1). The proposed protocol is for a 5-week intervention period that involves twice daily use of a myofunctional device called MyoMunchee  . There have been no previous trials using the MyoMunchee  for management of oral hygiene and dysphagia with residents of an aged care setting. However, the intervention protocol was based on previous research, a cluster randomized control trial utilized a myofunctional device to promote lip closure and nasal breathing for oral neuromuscular training for those 65 years and older in short term care units with impaired swallowing [18]. The results indicated that swallowing function significantly improved for those in the intervention group immediately following 5 weeks of therapy and a significant reduction in signs of aspiration 6 months post treatment was found compared to the control group.

**Fig. 1:**
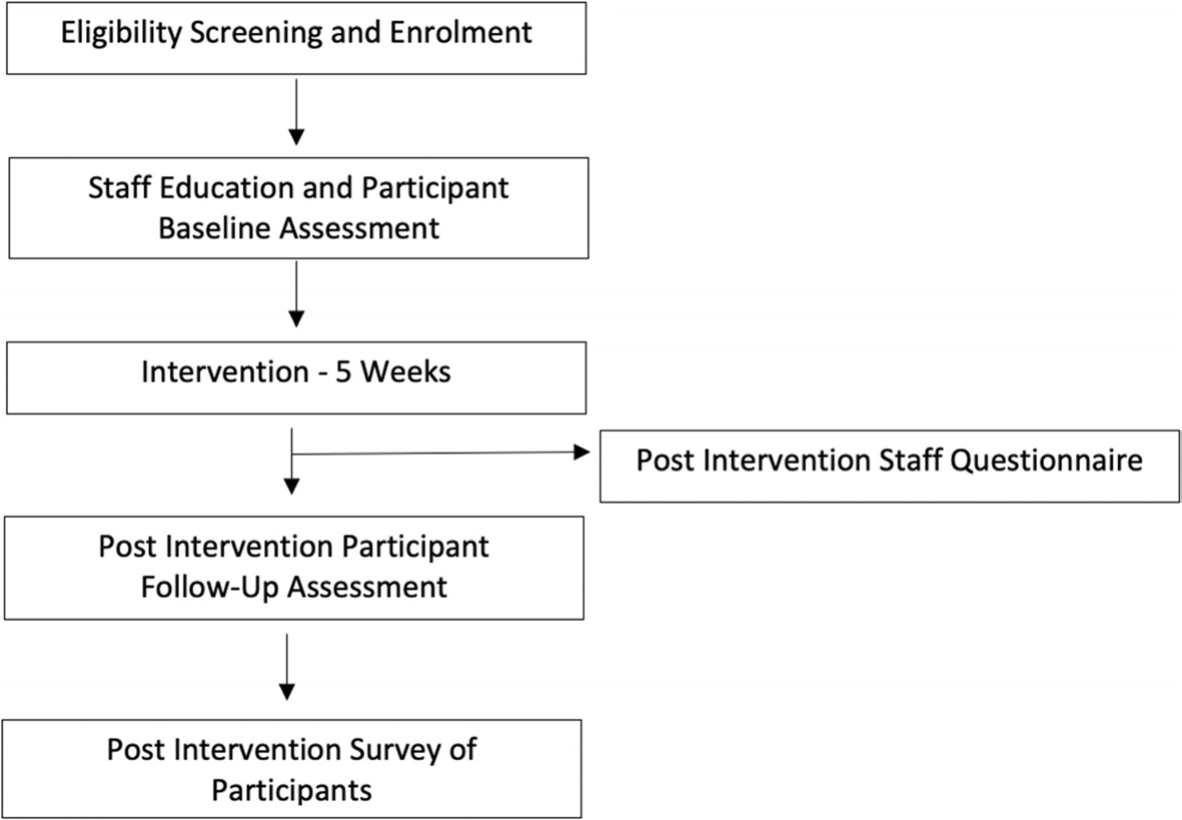
Flowchart of study procedures

If this trial is found to be feasible, the results will provide information for calculating the sample size needed for a randomized control trial [27, 28] as well as supporting the application of the device to more vulnerable aged populations. An overview of the study procedures is provided in Fig. 1.

### Primary aims

The primary aims of this study are as follows:

- Aim 1. Determine the feasibility of the use of myofunctional devices with an aged care population.
- Aim 2. Determine the feasibility of using a myofunctional device to improve oral hygiene, swallowing function, and/or dysphagia.

### Study setting and recruitment

This will be a single-intervention site study, conducted at an aged care facility (ACF) in Newcastle, Australia, with data analysis occurring at the University of Newcastle, Australia. Given the restrictions around COVID-19 in Australia, and specifically in the aged care sector, a decision was made for a single-site study to reduce the possibilities of contamination across multiple sites. The ACF consists of residents ranging in level of care including self-assisted retirement village living, residential care, low to high need dementia care, and palliative care.

Participants will be recruited with the assistance of care staff (nursing/allied health professionals) at the ACF, by the provision of recruitment flyers and participant information statements outlining the study to residents/legal guardians who meet the inclusion criteria.

All residents and care staff who meet the selection criteria will be eligible to participate in this study. They will be identified by the Nursing Unit Managers (NUM) and the speech pathologist/s at the ACF and provided with an information flyer. The contact details for the study team have been provided on all flyers and information statements inviting prospective participants/legal guardians to contact the study team prior to enrolment in the study.

Care staff will be assisting in providing the intervention as part of their routine care for residents but will be invited to participate in a post intervention questionnaire through study flyers placed in break rooms and following intervention education sessions by the study team. Staff will be provided with education and training in the use of myofunctional devices before the study commences. All care staff will be supervising and assisting with the intervention for residents who consent to the research, as part of their usual oral care routine. This is approved and directed by management at the trial site as part of their employee role. Ongoing supervision and support will be provided to care staff by nurse unit managers as well as the research team throughout the study. Care staff will be invited to participate in a post-intervention questionnaire regarding their experience of supervising and assisting residents with the trial intervention. While further detail may be gained from staff via other methods including post intervention focus groups, questionnaires were considered to be the most appropriate way of receiving feedback from staff. The decision by senior management of the facility was to ensure minimal disruption to care practices, as well as adherence to restrictions surrounding COVID-19. Care staff who wish to participate in the post-intervention questionnaire will need to complete a consent form and questionnaire which will be provided by the Nursing Unit Manager (NUM) of their ward.

The residents/families of residents and care staff will be provided with a participant information statement regarding the study and then followed up by the NUM/speech pathologist for interest in study participation. There will be an opportunity for residents, family of residents, and care staff to ask questions before and after consenting to participate. Questions regarding the study can be directed by the staff at the ACF to the Principal Investigator. Residents and care staff will be required to sign and provide written consent for participation. The capacity to consent for residents will be determined by nursing unit managers prior to approaching residents to participate in the study.

Explanation will be provided to residents/family of residents that their usual oral health care and dysphagia treatment will not vary with or without participation in this study. Further to this, a statement will be provided to participants that based on the results of the feasibility study, a larger clinical trial using the intervention may not be performed.

This research will involve the use of information without personal identifiers, and it will be obtained from individuals or gathered from medical files by care staff appointed by the ACF.

As this is a protocol for a feasibility study utilizing a myofunctional device not previously trialled in this setting, all consenting residents will receive the treatment intervention to better understand acceptability of the device and feasibility of completing a larger trial. No randomization of participants will occur. There is variation within the literature regarding the appropriate sample size for traditional feasibility studies, ranging from 10 to 75 participants [29]. The aim for this study is to recruit up to 50 participants. The targeted recruitment number of aged care residents and staff is based the on the available recruitment pool at the aged care facility, and the possible discontinuation rate within an aged care population, as well as staff availability due to varied shifts and leave due to COVID-19.

The expected period to recruit participants is 4 weeks. The timeframe for recruitment has been considered due to all residents being required to commence the treatment at the same time secondary to restrictions and access to the aged care facility during the COVID-19 pandemic. Staff training and education will be conducted face to face in small group sessions during the recruitment period by the research team.

### Participants

The participants will include a sample of up to 50 residents from the ACF and 10 care staff (nursing and/or allied health staff) who will oversee the daily use of the device during the treatment period.

Residents at the ACF who meet the following selection criteria and who consent to participating in the study will receive the treatment.

1. Age > 65 years
2. Ability to understand English and follow instructions for timed water swallow test and use of the myofunctional device
3. Residents receiving texture modified diets (including normal cut up, easy chew, minced moist, and puree diets) and/or fluids
4. Residents with natural teeth, dentures (partial and full), and edentulous

Residents will be excluded for the following reasons:

1. Inability to provide informed consent including diminished understanding or comprehension or inadequate English proficiency to follow directions for the intervention
2. On an end of life/palliative care pathway
3. Conditions that interfere with a patient’s ability to comply with all treatment(s) and procedure(s) and to follow study guidelines
4. Identified temporomandibular dysfunction
5. Identified by the visiting oral health professional to have tooth mobility

Care staff will be included if they are care staff approved by the ACF to participate in monitoring the intervention as part of their usual duties. Care staff will be excluded if they are not nursing or allied health professionals.

### Study intervention

Aged care resident participants will complete a 5-week intervention program using the MyoMunchee  . The device will be used twice daily (morning and evening recommended at the time of usual oral care routine) by the participants. This will require active use of the device with the action of chewing following the placement of the device in the oral cavity. The treatment has a gradual increase in the amount of time the device is used weekly to allow for adjustment to oral comfort as per the MyoMunchee  guidelines for device use [30] and previous research in oral neuromuscular training in those aged 65 years and older [18]. The duration for device use will start at 1 min twice a day in the first week and increase by 1 min each week, to 5 min twice a day by the fifth week of intervention (as seen in Table 1).

**Table 1:** Overview intervention schedule

Training and intervention	Content	Staff responsibility
Week 1	Device use twice daily for 1 min (morning and evening)	Supervision with device use and documentation of completion of intervention twice daily. This will continue from weeks 1–5.
Week 2	Device use twice daily for 2 min (morning and evening)	
Week 3	Device use twice daily for 3 min (morning and evening)	
Week 4	Device use twice daily for 4 min (morning and evening)	
Week 5	Device use twice daily for 5 min (morning and evening)	

Care staff will facilitate the delivery of the 5-week intervention, 7 days per week, with daily monitoring and twice daily documentation after each use of the device. Completed documentation of the device use will be collected and stored by care staff at the end of each of the 5 weeks of intervention. The research team will provide the residents/care staff with instructions and a guide for the use of the device with education regarding familiarization and instructions for use of the device (MyoMunchee  ), cleaning and storage of the device, and documentation of device use. Explanation regarding the cleaning, cleaning schedule, and storage of this will be provided as per MyoMunchee  cleaning protocol, which includes rinsing the device in water before and after use, shaking dry, and storing in the provided storage container.

### Treatment fidelity

Fidelity will be ensured by way of all care staff overseeing the intervention receiving the same training and education prior to the 5-week treatment. This training will be conducted by the same member of the research team who will also be conducting all pre- and post-treatment assessments with residents. Further fidelity checking will be conducted by the primary researcher. A random sample (approximately 10%) of participants daily treatment sessions will be observed by the primary researcher. The researcher will evaluate appropriate use of the device by participants and/or supported by care staff, as well as cleaning of device and accuracy of data recording.

### Treatment adherence

Twice daily use of the myofunctional device will be recorded by the care staff at the ACF via a checklist. An example of the first week of intervention checklist is provided in the appendix (Additional file 2). The nurse unit managers for each area of the ACF will ensure care staff are accurately documenting intervention completion. The daily checklist will be collected by the care staff at the ACF at the end of each week of intervention and replaced with a new checklist for the corresponding week of intervention which includes instructions on the duration of device use for that week. The study team will be available to answer questions from the care staff regarding the intervention during the 5 weeks and monitor for deviations from the study protocol such as the use of the device or adherence of the participant or the study site staff.

### Participant retention

The strategies used to maximize participant retention include education and training provided to care staff who will be responsible for facilitating the delivery of the intervention, and ensuring the intervention is completed twice daily for the specified duration. The participants will be provided with the device for the duration of the intervention and will also be permitted to retain the device following the intervention period.

### Safety monitoring

This study involves a new intervention with a vulnerable population. However, the intervention uses a device that has Australian Therapeutic Goods Approval and the residents who will be using this device will be monitored by care staff during the intervention as per Table 2.

**Table 2:** Potential risks and solutions for residents undertaking intervention

Potential risks	Solutions
Aspiration of saliva secondary to increased saliva production during device use.	Aspiration of saliva occurs daily. The continued use of usual oral care during this study to ensure adequate oral hygiene should assist with reducing complications that may arise from aspiration of saliva. Monitoring by care staff to ensure participants have adequate salvia management/control, such as monitoring for coughing/voice change with device use. If coughing occurs through the use of the device and increased saliva production, a referral will be made for review by the medical officer, as well as speech pathology at the ACF to review swallowing function (and appropriateness for continuation in the study).
Initial discomfort to gums/jaw with the action of chewing the device	The treatment intervention incorporates staged in time use of the device, increasing weekly to allow for adjustment to oral comfort. Supervision by care staff will be provided during the device use. If oral discomfort is identified/reported, a review by the researcher and speech pathologist at the ACF will take place and recommendations will be made regarding continuation in the study. Sizing and selection of the device have been considered for oral comfort and ease, placing, and removing the device from the oral cavity with an eternal tab/handle.
Infection control	Infection control procedures are outlined in the treatment manual and are consistent with COVID 19 safety and cleaning measures. Staff will be trained in cleaning protocols for the device (which are not aerosol generated) to reduce the risk of infection. Daily cleaning will be tracked with the completion of a daily check list by care staff at the ACF.

The risks to the participants’ undertaking intervention are considered negligible to low risk. There is low foreseeable risk of harm or discomfort [31]. There are no known risks for the care staff assisting with the intervention outside of usual oral care assistance.

Further considerations have been made regarding storage and labeling of the devices to minimize infection control risks (as per Table 3).

**Table 3:** Device considerations and storage

Potential concern	Strategy
Device labelling/identification	Devices will be stored as per dentures in storage container specifically for the MyoMunchee, labeled with the participant’s identification number. The MyoMunchee will be stored as per the ACF protocol for storage and identification of usual oral hygiene products.
Lost/misplaced device	In the event of the MyoMunchee being misplaced/lost a new replacement device will be provided

Data monitoring will occur throughout the study for the safety of residents undergoing the intervention via review of pre- and post-intervention assessment results, daily monitoring by care staff, and review of daily data sheets to prevent adverse events.

Monitoring and reporting of adverse events (AE), serious adverse events (SAE), and unexpected events (UE) will be conducted as per the ACF incident reporting system “ionMY” (governance, risk management, compliance platform) and entered by care staff as per the ACF incident reporting guidelines. Events will be reported to the principal investigator within 24 h of the UE/AE/SAE and entered into the University of Newcastle adverse events form in Research Information Management System within 72 h of being reported as per NHMRC guidelines [31]. All UE/AE/SAE will be assessed by the principal investigator using the University of Newcastle risk assessment matrix and NHMRC [31] guidelines of level of severity and reported to the sponsor and trial site by the principal investigator within 24 h of the principal investigator becoming aware of any urgent safety concerns.

Discontinuation of intervention for a participant may occur if an AE or medical condition occurs such that participation in the study would not be in the best interest of the participant or the participant met an exclusion criterion (newly developed or not identified on consent) that precludes further study participation.

### Assessments and measures

Determining the feasibility of the intervention by residents and care staff is needed to decide if there will be progression to a larger trial using the MD in other aged care settings and/or with more vulnerable populations. Feasibility will be determined by the acceptability of the device by residents and care staff, study recruitment and retention, and adherence to the intervention protocol. Acceptability will be measured by administering two surveys: to determine residents’ acceptability of the device and intervention and care staff acceptability in supporting residents during the intervention. Information will be obtained using both a 5-point Likert scale (very difficult – very easy), dichotomous questions (yes – no), and free text responses. An average positive rating (participants choosing the response easy/very easy/yes) of >70% across all parameters of acceptability will be needed to progress to a larger trial.

Study recruitment and retention will be measured by the proportion of consenting residents and those who complete the 5-week intervention. As this study is exploring the use of a MD with an aged care population, the criteria for progression to further trials will be determined by a resident consent rate of 30%, and a retention rate across the intervention of 70%.

Adherence to the intervention will be measured by total occasions of the device use at the 3-week (3 min of device use twice per day) and 5-week (5 min of device use twice per day) time point of the intervention. The collection of occasions of device use at three weeks is aligned with timeframes for existing recommendations for daily oral health care [32]. For this reason, the criteria for progression to a larger trial will consider both timepoint measurements of 85% adherence at 3 weeks and 70% adherence at 5 weeks of intervention.

The key feasibility outcomes and corresponding criteria for progression to a larger trial are outlined in Table 4.

**Table 4:** Feasibility outcomes and criteria for trial progression

Outcomes	Measurement	Criteria
**Acceptability** (average overall 70%)		
Resident ease of use	Very easy – very difficult	>70% of respondents chose the response easy or very easy/Yes
Resident oral comfort with device use	Very easy – very difficult	
Resident perceived changes to oral health and swallowing	Yes/unsure/no	
Care staff perception of resident’s ease of device use	Very easy – very difficult	
Care staff perception of perceived changes to resident’s oral health and swallowing	Yes/unsure/no	
Impact on care staff workload (time)	Yes/unsure/no	
**Study recruitment and retention**		
Recruitment consent rate	Proportion of eligible residents consented	>30%
Recruitment retention rate	Proportion of residents who completed the 5-week intervention	>70%
**Adherence to the intervention protocol** (both measures will be considered)		
Occasions of use after 3 weeks	Total number of device usage from 42 possible occasions	>85%
Occasions of use after 5 weeks	Total number of device usage from 70 possible occasions	>70%

Feasibility testing is also needed to determine what clinical outcome measures are sensitive enough to detect any change to oral care and swallowing function. Additional exploratory measures will be collected 1-week pre and post intervention, including measurements of oral health, aspiration risk, mastication ability, presence of dysphagia, functional oral intake, and self-perception of eating. Details of each of these measures are provided in Table 5.

**Table 5:** Secondary outcome measures for collection 1-week pre- and 1-week post-intervention

Outcome measure	Reference	Outcome	Description
Oral Health Assessment Tool (OHAT)	[33]	Oral health	Reliable and valid screening tool for use in aged care and with cognitive impairment; Approximately 7–8 min to administer; 8 items, Rating scale – 0 = healthy, 1 = changes, 2 = unhealthy; Total score out of 16. The higher the score the worse the oral health; Items that score 1 indicate intervention is required, and items scoring 2 indicate referral to a dental professional is required
Timed Water Swallow Test (TWST)	[34]	Aspiration risk	Swallow speed is a sensitive indicator for identifying patients at risk of swallow dysfunction; Choking in 100ml WST may be a potential indicator for follow up aspiration; Measures swallow time, number of swallows and observes for signs of choking; Abnormal swallow is defined as a speed below 10ml/s (amount of water divided by elapsed time); Count the number of swallows taken to consume 100mls water; Time taken to consume 100mls water
Test of Mastication and Swallowing Solids (TOMASS)	[35]	Mastication ability	Quantitative assessment of solid bolus ingestion; Sensitive in detecting changes in performance ability of mastication; High interrater and test-retest reliability; Count number of bites, number of masticatory cycles per bite, number of swallows per bite; More likely to identify patients with subtle oral phase impairment or bolus transition issues; Normative ranges in older adults: number of bites (male 1.47/female 1.87), time in seconds (male 32.61/female 41.85), total number of swallows (male 3.61/female 3.5), masticatory cycle (male 37.6/female 41.65)
Mann Assessment of Swallowing Ability (MASA)	[36]	Identify swallowing disorders	Screening bedside tool to identify eating and swallow disorders in stroke and other diseases; Used to quantify aspiration risk; 24 clinical items; 4 components of the assessment include, general patient examination, oral preparation, oral phase, and the pharyngeal phase; 5–10 point rating scale; score out of /200, >178 = normal, 168–177 = mild, 139–167 = moderate, <138 = severe; risk of aspiration is defined on a sum of the 4 scores/categories, >170 = normal, 149–169 = mild, 141–148 = moderate, <140 = severe
Functional Oral Intake Scale (FOIS)	[37]	Functionality	7-point ordinal scale; Functional level of oral intake of food and liquid; Interrater reliability high and sensitive to changes; Levels 1–3 relate to non-oral feeding; Levels 4–7 relate to varying degrees of oral feeding; All levels focus on what is/not consumed orally
Eating Assessment Tool (EAT-10)	[38]	Self-perceived symptoms	Screen self-perceived oropharyngeal dysphagia symptoms: Scores range from 0 to 40; Scores >3 is indicative of dysphagia; 10 questions rated on a 5-point scale, 0 = no problem, 4 = severe problem; Scores >15 indicative of aspiration risk; An elevated EAT-10 score indicates a higher self-perception of dysphagia

### Data collection

The number of residents approached to participate in the study versus those who consented to participate will be collected in the recruitment period before pre-intervention assessments commence (as per Table 4). The intervention completion rate of those who consented to participate and those who completed the 5-week intervention will be collected following the 5-week intervention and collection of the post-intervention assessment. Data will be collected for adherence to the intervention during the 5-week study period by way of daily checklist completion by the care staff that residents have completed the intervention twice daily for the specified amount of time. The checklist will be collected at the end of each week and entered into an electronic database by the principal investigator. Further to this, a survey of the residents completing the intervention, as well as a questionnaire for the care staff assisting participants with the intervention will be taken at the completion of the 5-week intervention trial. Assistance in reading the survey for residents with visual impairment and aphasia will be provided by a staff member at the ACF.

Pre- and post-intervention outcome measures will be collected for comparison to observe changes in oral hygiene and swallowing function. Data collection of outcome measures will be completed by a qualified clinical speech pathologist/principal investigator (with over 17 years clinical experience). Reliability checking of pre- and post-outcome measure collection of 10% of residents will occur onsite at the ACF by a qualified clinical speech pathologist from the University of Newcastle, independent of the study.

**Fig. 2:**
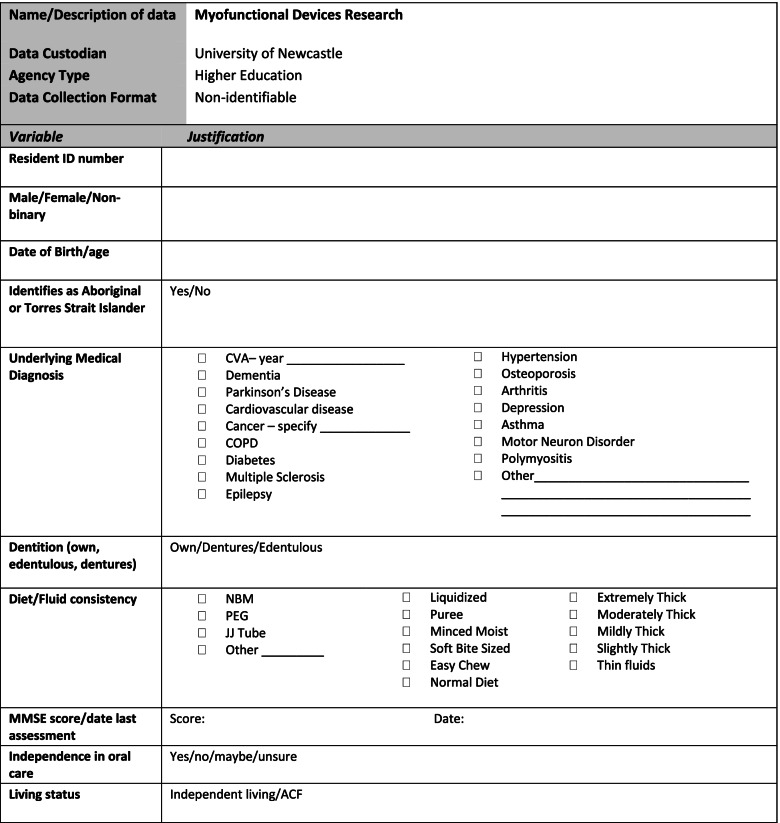
Resident data collection form

Resident participant’s demographic data (see Fig. 2.) will be extracted from the medical files of each consenting participant by an employee appointed by the ACF. The data will be deidentified using a code and then provided to the research team.

### Data analysis

A sample size calculation is not provided for this feasibility study. Rather, it is estimated that 50 participants will be recruited over a 1-month period. This sample size is based on (1) a proportion of eligible residents and care staff at the aged care facility who are able to consent, and (2) an audit of 79 pilot and feasibility trials which indicated a median target sample size of 36 (IQR 25–50) is recommended for feasibility studies [39]. Assessment of consenting rates versus intervention completion rates will involve statistical analyses and will be performed using SPSS, version 22. The primary outcome will be reported as numbers and percentages. Outcome measures will be summarised using mean (SD) for normally distributed data, median (interquartile range) for non-normally distributed data, and number (percent for categorical data). Pre- and post-intervention comparisons will be performed using a paired *t* test.

Data collected through post-intervention survey of residents and care staff questionnaires will use qualitative content analysis as described by Graneheim and Lundman [40] to analyze participants’ free text responses. Free text data will be transcribed verbatim and analyzed with NVivo 12.0 Software to assist with the identification of patterns in the text segments of the care staff questionnaire. For questions using a Likert scale data will be analyzed descriptively using distribution of responses provided by participants.

## Discussion

The link between oral health and the inability to manage this independently is a known predictor of aspiration pneumonia in vulnerable populations such as the elderly and chronically ill [19]. The links between poor oral health with systemic disease, morbidity, and mortality has been demonstrated extensively throughout allied health, nursing, and dental literature [5]. If oral health is reduced, people are not only at greater risk of aspiration pneumonia, but they may have pain or poorer dentition which impacts on mastication and swallowing, and reduced oral intake, with potential for malnutrition, poorer health outcomes, and reduced quality of life [20].

The results of Shortland and colleagues [15] systematic review reported improvements in oral hygiene, oral behaviors, and swallowing, along with breathing and speech to be associated with the use of myofunctional devices. Therefore, it would be relevant to further explore treatment dosage, utilization, and outcomes of a myofunctional device in a population such as the elderly who make up a large proportion of those whose oral health and swallowing function may be impacted [21].

The outcomes from this study of feasibility and efficacy for the use of a myofunctional device in improving in oral health and dysphagia in an aged care population will further guide the design of a randomized control trial.

## Supplementary Information

**Additional file 1.** Myofunctional device use in oral care and swallowing in an aged care population: A feasibility study.

**Additional file 2.** Protocol. Week 1.

## Data Availability

The study will adhere to The Australian Code for the Responsible Conduct of Research [39] as well as the University of Newcastle Data management policy [40]. Following the completion and publication of the feasibility study the datasets used and analyzed will be available from the author on reasonable request.

## Abbreviations

ACF: Aged Care Facility
OHAT: Oral Health Assessment Tool
WST: Timed Water Swallow Test
TOMASS: Test of Mastication and Swallowing Solids
MASA: Mann Assessment of Swallowing Ability
FOIS: Functional Oral Intake Scale
EAT-10: Eating Assessment Tool
MMSE: Mini Mental State Examination
NUM: Nursing Unit Manager
AE: Adverse events
SAE: Serious adverse events
UE: Unexpected events
PIS: Participant information statement
AIHW: Australian Institute of Health and Welfare
AHMAC: Australian Health Ministers Advisory Council
