# Supplementary material for: Myofunctional device use in oral care and swallowing: a protocol for a feasibility study in an aged care population
Source: Pilot Feasibility Stud. 2022 Aug 19;8:187. doi: 10.1186/s40814-022-01148-3 (PMC9388991; doi:10.1186/s40814-022-01148-3)
Supplement: Supplementary file 1 — Additional file 1. Myofunctional device use in oral care and swallowing in an aged care population: A feasibility study. [file 40814_2022_1148_MOESM1_ESM.docx]

**RESEARCH PROTOCOL**

***Full Title: Myofunctional device use in oral care and swallowing in an aged care population: A feasibility study***

**STUDY INVESTIGATOR(S)**

# Principal Investigator(s):

| Hollie-Ann ShortlandPhD Candidate – Speech PathologyUniversity of NewcastleUniversity Drive, CallaghanNSW,AustraliaPh. +61 249217347Email: [Hollie-Ann.Shortland@newcastle.edu.au](mailto:Hollie-Ann.Shortland@newcastle.edu.au)*Role: study design, training, assessment, analysis* | A/Prof Sally Hewat Speech Pathologist  University of Newcastle University Drive, CallaghanNSW, Australia  Ph. +61 249215159  Email: [Sally.Hewat@newcastle.edu.au](mailto:Sally.Hewat@newcastle.edu.au) |
| --- | --- |

# Co - Investigator(s):

| A/Prof Anne Vertigan Director of Speech Pathology  John Hunter Hospital  Lookout Road, New Lambton  NSW,  Australia  Ph. +6149213726  Email: [Anne.Vertigan@health.nsw.gov.au](mailto:Anne.Vertigan@health.nsw.gov.au) | Dr Gwendalyn Webb Speech Pathologist  University of Newcastle University Drive, CallaghanNSW, Australia  Ph. +61 249215694  Email: [Gwendalyn.Webb@newcastle.edu.au](mailto:Gwendalyn.Webb@newcastle.edu.au) |
| --- | --- |

**OTHER PERSONNEL**

| Maroba Aged Care Edith St, Waratah  NSW,  Australia  Ph. +61 49350300 |
| --- |

# ABSTRACT

**Background:** Oral health and the inability to manage this independently is a known predictor of aspiration pneumonia in vulnerable populations such as the elderly and chronically ill, with the link between poor oral health with systemic disease, morbidity and mortality demonstrated extensively throughout allied health, nursing, and dental literature. If oral health is reduced, people are not only at greater risk of aspiration pneumonia, but they may have pain or poorer dentition which impacts on mastication and swallowing, and reduced oral intake, with potential for malnutrition, poorer health outcomes and reduced quality of life.

There are currently limited oral care protocols with measurable outcomes that are used in hospitals and care facilities in Australia. This is of concern to speech pathologists due to the link between poor oral care and the potential for aspiration in vulnerable populations such as the elderly. However, a recent literature review by Shortland and colleagues (2021) reported improvements in oral hygiene, oral behaviours and swallowing, along with breathing and speech have been found to be associated with the use of myofunctional devices.

**Methods/Design:** This project is a feasibility study for a new five-week intervention for oral hygiene and dysphagia for residents 65> years old in an aged care setting. Feasibility will be measured through the acceptability of the treatment and measured by number of consenting participants, trial completion rates, treatment adherence, and staff implementation. Efficacy will be determined by secondary outcome measures through pre and post assessment of oral health, perceived dysphagia symptoms and presence of dysphagia.

**Discussion:** The results of this trial will provide important information regarding the acceptability and feasibility of a myofunctional device in improving oral care and dysphagia in elderly patients in an aged care facility. This knowledge will further guide, and inform design of a larger trial or future research.

# KEY WORDS

Myofunctional Device, Dysphagia, Oral Hygiene

# ADMINISTRATIVE INFORMATION

| Title | Myofunctional device use in oral care and swallowing in  an aged care population: A pilot feasibility study |
| --- | --- |
| Trial registration | Registration submitted with Australian New Zealand Clinical Trials Registry |
| Protocol version | 11^th^ July 2021 Version 1.0 |
| Funding | Provision of the myofunctional device has been provided by MyoMunchee |
| Author details | - Hollie-Ann Shortland – University of Newcastle, Australia - Associate Professor Sally Hewat - University of Newcastle, Australia - Associate Professor Anne Vertigan – Hunter New England Health/ University of Newcastle, Australia - Dr Gwendalyn Webb - University of Newcastle, Australia |
| Name and contact information for the trial sponsor | University of Newcastle, Speech Pathology  A/Prof Sally Hewat (Primary supervisor and chief investigator) |
| Role of sponsor | To oversee the implementation of the trial, monitor trial progress, and ensure it adheres to ethical requirements, and supervision of HDR student coordinating the trial.  Funding for the HDR scholarship has been provided by MyoMunchee, who have provided a 3.5 year Higher Degree of Research scholarship grant, awarded to the Chief Investigator (A/Prof. Sally Hewat) and to one of the two co-supervisors (Dr Gwendalyn Webb). |

# BACKGROUND AND SIGNIFICANCE

The link between poor oral health with systemic disease, morbidity and mortality has been demonstrated extensively throughout allied health, nursing, and dental literature (Danckert, Ryan, Plummer & Williams, 2016; Murray & Scholton, 2017; Ajwani, Jayanti, Burkolter, Anderson, Bhole, Itaoui & George, 2017; Wang, Huang, Chou & Yu, 2015).

Oral health is important in both children and adults. Poor oral health, such as tooth decay, gum disease and tooth loss, contributed to 4.5% of non-fatal diseases in Australia in 2015, with 78% of oral disorders in people aged 85 years and over (Council of Australian Government Health Council (COAG) 2019). Oral health deteriorates over a person’s lifetime, with children aged 5-10 years of age on average having 1.5 decayed, missing or filled teeth. In contrast, an adult 75+ years on average has 24.4 decayed, missing or filled teeth (Australian Institute of Health and Welfare (AIHW) 2021). The rate of tooth decay has been reported to be higher in the Indigenous population and those in rural and remote areas in Australia (Australian Health Ministers Advisory Council (AHMAC), 2017). Early stage gum disease, known as gingivitis, is caused by an accumulation of plaque on the teeth and gum line. It can cause inflammation and irritation to the gums which if left untreated leads to more serious periodontal disease, including damage to the soft tissue and loss of teeth (Bourke, n.d). In 2017-2018, the proportion of adults with advanced stage gum disease, periodontitis, increased with age from 51% in those 55-74 years, to 69% in those 75 years and over (COAG, 2019). Further to this, the AIHW (2021) reported that between 2017-2018 there were approximately 72,000 hospitalisations for dental conditions that may have been prevented with earlier treatment.

Factors that influence poor oral health are consumption of sugar, alcohol and tobacco; reduced access to dental services; and reduction in good oral hygiene (AIHW, 2021). The AHMAC identified four population groups that are at greater risk of poor oral health, including socially disadvantaged or low income; Aboriginal and Torres Strait Islander Australians; Those living in rural and remote areas; and people with additional and or specialized health care needs such as those with mental illness or the frail older population.

Reduced oral health and an inability to manage oral heath independently is a predictor of aspiration pneumonia in vulnerable populations such as the elderly and chronically ill (Langmore, Terpenning, Schork, Chen, Murray, Lopatin & Loesche, 1998). If oral health is reduced, not only are people at greater risk of aspiration pneumonia, they may have pain or poorer dentition. This impacts on mastication and swallowing, with potential for malnutrition, poorer health outcomes and reduced quality of life (Luong, Drysdale, Campbell, deClifford, Lam, Coutsouvelis, Danckert, 2018). Reduced oral health is associated with a number of chronic diseases including stroke and cardiovascular disease (Slade, Spencer, Roberts-Thompson, 2007).

There are several factors that impact in oral hygiene and swallowing ability. An oral breathing pattern may result in a dry oral cavity and reduced oral hygiene, which further impacts on swallowing and may increase the risk of aspiration pneumonia (Jayanti, 2006). Reduced oral hygiene may lead to pain, increased difficulties with mastication, further leading to decreased muscle bulk and poor tolerance of diet consistency (Jayanti, 2006). The need for change of diet consistency may be influenced by the risk of aspiration or choking, and in populations such as the elderly these diet changes may lead to increased risk of malnutrition, dehydration and reduction of quality of life (Hagglund et al., 2019). A study by Jayanti (2006) explored the impact of myofunctional devices on speech, swallowing and quality of life in the elderly. They reported a reduction in lip tone in the elderly population may further influence an oral breathing pattern over nasal breathing. In their scoping review of integrated oral care for stroke patients, Ajwani and colleagues (2017) reported an overwhelming lack of knowledge in oral healthcare by nursing staff in Australia. Their scoping review of the literature highlighted the importance of oral health post stroke in reducing risk of aspiration pneumonia, the need for integrated oral health programs, and also discussed the need for maintaining optimal oral health due to the links between gingivitis and cerebrovascular infarction as well as periodontal disease and stroke.

Currently there are limited oral care protocols with measurable outcomes that are used in hospitals and care facilities in Australia (Drapal, 2015). These protocols have been described as being ad-hoc and often not prioritised in patient care (Drapal, 2015). This is of concern to speech pathologists due to the impact of poor oral health on mastication, swallowing function and increased risk of aspiration. However, a recent literature review (Shortland, Hewat, Vertigan & Webb, 2021) found that improvements in oral hygiene, oral behaviours and swallowing, along with breathing and speech have been found to be associated with the use of orofacial myofunctional therapy and myofunctional devices (Korbmacher, Schwan, Berndsen, Bull, & Kahl-Nieke, 2004).

Orofacial myofunctional therapy is the treatment of the muscles of the face and mouth with the purpose of correcting orofacial functions (Farrell & Darcy, 2018). Orofacial myofunctional therapy has been utilized since the 1900’s to assist with orofacial dysfunction including, mandibular growth, malfunctions of the tongue, abnormal swallowing, nasal breathing and facial appearance (Farrell et al., 2018). This treatment aims to improve orofacial functions such as lip seal, mastication, swallowing, nasal breathing and oral behaviours (Wishney, Darendeliler, & Dalci, 2019). Similarly, myofunctional devices have been utilized for decades in orthodontic treatment of orofacial dysfunction (Wishney, et al., 2019). Historically, the literature regarding myofunctional devices has predominantly been in the areas of malocclusion and obstructive sleep apnoea, (Wishney et al, 2019, and Huang, Chuang, Hervy-Auboiron, Paiva, Lin, Guilleminault, 2018) but has also shown positive outcomes in sleep disordered breathing (Moeller, Paskay, Gelb, 2014 and Huang et al, 2018) and cranio-facial muscle competence (Bourke, n.d.).

The past 30 years have seen an increase in orthodontic/dental, respiratory and sleep medicine, and otolaryngology literature supporting the use of orofacial myofunctional therapy, and more specifically prefabricated myofunctional devices in the treatment of orofacial dysfunction. In a study by Shortland and colleagues (pending publication) into speech pathologists’ use and outcomes of myofunctional devices in therapy programs, there was found to be both similarities and differences in the use of myofunctional devices, and the therapy programs in which these devices are incorporated. This varied across intervention areas, caseloads and diagnoses amongst speech pathologists who utilized them. Shortland and Colleagues (awaiting publication) reported the type of myofunctional device, timing of introduction, utilisation in isolation, adherence, and dosage variation of myofunctional devices used contributed to successful outcomes for swallowing, oral hygiene, breathing and speech. However further education and research into myofunctional device use and guidelines to further direct their use in speech pathology intervention was recommended, along with a coordinated approach and team input in assessment and intervention.

Despite this increase of evidence, there continues to be limited research that exists in speech pathology that addresses the use and outcomes of myofunctional devices in clinical practice (Shortland and colleagues, 2021). The potential impact of improvement on orofacial function, including oral hygiene and swallowing, has already been identified with the use of myofunctional devices in literature from various health disciplines (Hägglund, Hägg, Wester, & Levring Jäghagen, 2019). Along with the link between reduced oral hygiene and aspiration pneumonia (Huang, Chiou, & Liu, 2017), and the impact of a reduction in oral hygiene on quality of life (Manabe, Teramoto, Tamiya, Okochi, & Hizawa, 2015). It would be relevant to further explore treatment dosage, utilisation and outcomes of a myofunctional device in a population such as the elderly who make up a large proportion of those whose oral health and swallowing function may be impacted on (Santoro, eSivla, Cardoso, Dias, & Beresford, 2011).

Further information is needed by way of a study to analyse feasibility prior to performing a randomised control trial, by testing procedures of myofunctional device use for acceptability, recruitment and retention of participants, and further determine sample size for a larger clinical trial to evaluate the use and outcomes of a myofunctional device in oral care and dysphagia treatment in an adult population in care facilities.

# AIM OF STUDY / RESEARCH QUESTION

The objective of this study is to assess the feasibility and efficacy of a using a myofunctional device to improve oral care and swallowing function in an aged care population.

# STUDY DESIGN/METHODOLOGY

The protocol presented is based on both the Standard Protocol Items: Recommendations For Interventional Trials (SPIRIT) (2013) and Consolidated Standards of Reporting Trials (CONSORT) (2018) guidelines, to conduct an acceptability and feasibility pilot study. The proposed protocol is for a new intervention. There have been no previous trials using the MyoMunchee
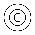
 (a myofunctional device) in the area of oral hygiene and dysphagia with those in an aged care setting. A cluster randomised control trial in Sweden by Hagglund and colleagues (2019) utilised a myofunctional device (used for promoting lip closure and nasal breathing) for oral neuromuscular training for those 65 years and older in short term care units with impaired swallowing. Hagglund and colleagues (2019) study indicated that swallowing function significantly improved for those in the intervention group immediately following 5 weeks of therapy with the use of the myofunctional device, and a significant reduction in signs of aspiration compared to the control group 6 months post treatment.

The use of this study will provide important information regarding the feasibility and efficacy of a new treatment for oral hygiene and dysphagia with the MyoMunchee
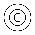
, and provide information for calculating the sample size needed for a randomised control trial (In, 2017, & Eldridge, et al., 2016). Thabane and Lancaster (2019), In (2017), and Eldridge, et al., (2016) highlight the importance of feasibility studies to further guide and inform conduct and design of a larger clinical trial or future research.

The feasibility of the treatment intervention will be measured by way of:

- Verbal survey of the residents receiving the intervention regarding experience using the device, using a 5-point Likert scale (very difficult – very easy), and dichotomous questions (yes – no) (Appendix A).
- Questionnaire for care staff (nursing/allied health) in the implementation and acceptability of assisting participants with the myofunctional device, using both a 5-point Likert scale (very difficult – very easy), dichotomous questions (yes – no), and free text (Appendix B).
- The consenting rates,
- Treatment completion rates,
- Treatment adherence (which will be monitored on the daily check list provided by care staff, see Appendix C, D, E, F, G).

Efficacy will be measured through the collection of pre and post intervention outcome measures using validated instruments to measure perceived dysphagia symptoms, and the presence of dysphagia. These outcome measures include:

- Oral Health Assessment Tool (OHAT) (Chalmers, Johnson, Tang, Titler, 2004) (Appendix H)
- Timed Water Swallowing Test (TWST) (Hughes &Wiles, 1996) (Appendix I)
- Test of Mastication and Swallowing Solids (TOMASS) (Huckabee, et al., 2018) (Appendix J)
- Mann Assessment of Swallowing Ability (MASA) (Mann, 2002) (Appendix K)
- Eating Assessment Tool (EAT-10) (Belafsky, et al., 2008) (Appendix L)
- Mini Mental State Examination (MMSE) (Malloy & Clarnette, 1999) (Appendix M)
- Functional Oral Intake Scale (FOIS) (Cary, Mann, & Groher, 2005) (Appendix N)

# STUDY SETTING/LOCATION

This will be a single intervention site study, conducted at Maroba Aged Care, Newcastle, Australia, with data analysis occurring at the University of Newcastle, Australia.

1. **Sites**

This study will involve recruitment of residents and care staff from Maroba Aged Care and will be conducted by researchers from the University of Newcastle, with the assistance of care staff (nursing/allied health) at Maroba Aged Care. Data once collected will be stored on a secured drive at the University of Newcastle for analysis.

# STUDY POPULATION AND SETTING

Eligible residents at Maroba Aged Care who meet the selection criteria outlined below and who consent to participating in the study will receive intervention.

Care staff (nursing/allied health) who consent to participate in the study will be responsible for overseeing the intervention of residents who are consented to the study.

# ELIGIBILITY CRITERIA

Residents:

**Inclusion criteria**

1. age > 65 years

2. Ability to understand English and follow instructions for timed water swallow test and use of the myofunctional device

3. Residents receiving texture modified diets and/or fluids

4. Residents with natural teeth, dentures (partial and full), and edentulous

**Exclusion criteria**

1. Circumstances that interfere with the participant’s or guardian’s ability to give informed consent, for example, diminished understanding or comprehension, or a language other than English spoken.

2. Those who are on an end of life/palliative care pathway

3.Conditions that interfere with a patient's ability to comply with all treatment(s)/procedure(s) or follow study guidelines (such as behavioural issues as a result of Dementia)

4. Identified Temporomandibular Dysfunction

5. Those who are identified by the visiting oral health professional to have tooth mobility

Care staff (inclusive of nursing and allied health):

**Inclusion criteria**

1. Care staff at Maroba Aged Care

2. Care staff approved by Maroba Aged Care to participate in the study

**Exclusion criteria**

1. Staff without nursing or allied health trained qualification

# STUDY OUTCOMES

1. **Quantitative**
2. **Primary Outcome**

Feasibility and acceptability of treatment intervention. This will include the number of participants approached versus consented, completed treatment rates, and adherence to the intervention (monitored by care staff with supervision of the intervention, and completion of daily intervention sheets).

1. **Secondary Outcome(s)**

Secondary outcome measures will be collected both pre and post intervention, and are outlined in Table 1.

**Table 1** Secondary outcome measures for collection pre and post intervention

| Outcome Measure | Reference | Outcome | Description |
| --- | --- | --- | --- |
| Oral Health Assessment Tool (OHAT) | [6] | Oral health | Reliable and valid screening tool for use in aged care and with cognitive impairment; Approximately 7-8 minutes to administer; 8 items, Rating scale – 0 = healthy, 1 = changes, 2 = unhealthy; Total score out of 16, The higher the score the worse the oral health; Items that score 1 indicate intervention is required, and items scoring 2 indicate referral to a dental professional is required |
| Timed Water Swallow Test (WST) | [18] | Aspiration risk | Swallow speed is a sensitive indicator for identifying patients at risk of swallow dysfunction; Choking in 100ml WST may be a potential indicator for follow up aspiration; Measures swallow time, number of swallows and observes for signs of choking; Abnormal swallow is defined as a speed below 10ml/s (amount of water divided by elapsed time); Count the number of swallows taken to consume 100mls water; Time taken to consume 100mls water |
| Test of Mastication and Swallowing Solids (TOMASS) | [17] | Mastication ability | Quantitative assessment of solid bolus ingestion; Sensitive in detecting changes in performance ability of mastication; High interrater and test-retest reliability; Count number of bites, number of masticatory cycles per bite, number of swallows per bite; More likely to identify patients with subtle oral phase impairment or bolus transition issues; Normative ranges in older adults: number of bites (male 1.47/female 1.87), time in seconds (male 32.61/female 41.85), total number of swallows (male 3.61/female 3.5), masticatory cycle (male 37.6/female 41.65) |
| Mann Assessment of Swallowing Ability (MASA) | [24] | Identify swallowing disorders | Screening bedside tool to identify eating and swallow disorders in stroke and other diseases; Used to quantify aspiration risk; 24 clinical items; 4 components of the assessment include, general patient examination, oral preparation, oral phase, and the pharyngeal phase; 5-10 point rating scale; score out of /200, >178 = normal, 168-177 = mild, 139-167 = moderate, <138 = severe; risk of aspiration is defined on a sum of the 4 scores/categories, >170 = normal, 149-169 = mild, 141-148 = moderate, <140 = severe |
| Functional Oral Intake Scale (FOIS) | [8] | Functionality | 7 point ordinal scale; Functional level of oral intake of food and liquid; Interrater reliability high and sensitive to changes; Levels 1-3 relate to non-oral feeding; Levels 4-7 relate to varying degrees of oral feeding; All levels focus on what is/not consumed orally |
| Eating Assessment Tool (EAT-10) | [4] | Self-perceived symptoms | Screen self-perceived oropharyngeal dysphagia symptoms; Scores range from 0-40; Scores >3 are indicative of dysphagia; 10 questions rated on a 5 point scale, 0 = no problem, 4 = severe problem; Scores >15 indicative of aspiration risk; An elevated EAT-10 score indicates a higher self-perception of dysphagia |
| Mini Mental State Examination (MMSE) | [26] | Cognitive function | Widely used screening test of mental function; Evaluates cognitive impairment in older adults; 10 minutes to complete; normal = 30-25, mild = 24-21, moderate = 21-10, severe = 9-0 |

1. **Qualitative**
2. Administer Care Staff Acceptability Questionnaire to those assisting with the intervention/observing the use of the myofunctional device to rate the ease of use and cleaning of the device, the impact on workload, and perceived acceptance by residents.

Administer a Resident Acceptability Survey to all enrolled residents, regarding ease of use, comfort with use, and if there were perceived changes to oral health or swallowing following the use of the device. Assistance in reading the survey to those residents with visual impairment, aphasia, and cognitive and intellectual impairment will be provided by the care staff.

# STUDY PROCEDURES

1. **Recruitment of participants**

All residents and care staff who meet the selection criteria will be eligible to participate in this study. They will be identified by the Nursing Unit Managers (NUM) and the speech pathologist/s at Maroba Aged Care and provided with an information flyer (Resident Information Flyer, Appendix O. Care Staff Information Flyer, Appendix P). The residents/families of residents/care staff will be provided with a participant information statement (Residents, Appendix Q. Care staff, Appendix R) regarding the study, and then followed up by the NUM/speech pathologist for interest in study participation. There will be an opportunity for residents, family of residents, and care staff to ask questions before and after consenting to participation. Questions regarding the study can be directed by the staff at Maroba Aged care to the Principal Investigator. Residents/family of residents/care staff will be required to sign and provide written consent for participation (Residents, Appendix S. Care staff, Appendix T). Consideration and return of consent for participation in the study will be 7 days after initial contact.

Explanation will be provided to residents/family of residents that their usual oral health care and dysphagia treatment will not vary with or without participation in this study. Further to this, a statement will be provided to participants that based on the results of the pilot study, the larger clinical trial may not be performed.

This research will involve the use of information without personal identifiers and it will be obtained from individuals or gathered from medical files by care staff appointed by Maroba Aged Care. The existing data information seen in section 9d. will be collected for each consented resident.

As this is a feasibility study utilising a myofunctional device not previously trialled in this setting, all consented residents will receive the treatment intervention to better understand acceptability of the device and feasibility of completing a larger trial. No randomisation of participants will occur. The number of participants that will be aimed for recruitment – up to 50 residents and 10 staff. This is based on the available recruitment pool, and the number of residents who may not give consent. Because this is a feasibility study this does not require sample size base.

The period of time for expected time to recruit participants is four weeks.

1. **Study procedures**

Staff:

Education will be provided before the commencement of the trial intervention by the principal investigator from the University of Newcastle to care staff, regarding familiarization and instructions for use of the myofunctional device (MyoMunchee
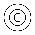
), cleaning and storage of the device, and documentation of device use.

The use of a myofunctional device (MyoMunchee
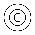
) will be required for each participant for the duration of this intervention and will be provided in kind by MyoMunchee
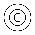
. Further explanation regarding the cleaning, cleaning schedule and storage of this will be provided in the treatment protocol as per MyoMunchee
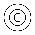
 cleaning protocol.

The research team will provide the residents/care staff with instructions and a guide for the use of the device. This will require active use of the device with the action of chewing.

The use of the myofunctional device will be monitored by the care staff at Maroba Aged Care, with documentation on a daily checklist provided for each participant. The daily checklist will be collected by the care staff at Maroba Aged Care at the end of each week of intervention and entered into an electronic database each week by the principal investigator.

Following the five week intervention, the care staff will be asked to complete a questionnaire regarding acceptability and feasibility of the trial intervention.

Residents:

Once participants who meet the selection criteria are enrolled in the study, outcome measures will be collected and stored by the principal investigator from the University of Newcastle. The collection of outcome measure should take approximately 60 minutes for each participant.

There will not be any alteration to the participant’s usual intervention for oral care or swallowing treatment unless this is deemed appropriate by the usual treating speech pathologist/s at Maroba Aged Care, or the treating medical/nursing team.

The trial intervention using the myofunctional device will commence within two weeks post pre-treatment outcome measure collection.

The myofunctional device (MyoMunchee
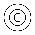
) will be used twice daily (morning and evening recommended at the time of usual oral care routine) by the participants. The duration for device use will start at 1 minute twice a day in the first week, and increase to 2 minutes twice a day in the second week, 3 minutes twice a day in the third week, 4 minutes twice a day in the fourth week, and 5 minutes twice a day use in the fifth week of intervention.

Following the five week trial of intervention, post-treatment outcome measures will be taken by the lead researcher from the University of Newcastle.

Post treatment intervention, residents will be asked to take part in a survey regarding the acceptability of the myofunctional devices.

Protocol Flow:

1. **Methods of data collection**

Data collection will be completed by a qualified clinical speech pathologist/principal investigator (with over 17 years clinical experience) with reliability checking of 10% of residents onsite at Maroba Aged Care by a qualified clinical speech pathologist from the University of Newcastle.

Pre and post treatment outcome measures will be taken for comparison, along with data collected for adherence to treatment by way of daily checklist completion by the care staff, which will be collected at the end of each week and stored by the research team for the duration of the trial. Further to this a survey of the residents completing the intervention, as well as a questionnaire for the care staff assisting participants with the intervention will be taken at the completion of the five week intervention trial.

1. **Access to Existing Data**

Data (as per the form below) will be extracted from the medical files of each consenting participant by an employee appointed by Maroba Aged Care. The data will be deidentified using a code and then provided to the research team.

| **Name/Description of data** | **Myofunctional Devices Research** | | | | |
| --- | --- | --- | --- | --- | --- |
| **Data Custodian** | University of Newcastle | | | | |
| **Agency Type** | Higher Education | | | | |
| **Data Collection Format** | Non-identifiable | | | | |
| ***Variable*** | ***Justification*** | | | | |
| **Resident ID number** |  | | | | |
| **Male/Female/Non-binary** |  | | | | |
| **Date of Birth/age** |  | | | | |
| **Identifies as Aboriginal or Torres Strait Islander** | Yes/No | | | | |
| **Underlying Medical Diagnosis** | - CVA– year _________________ - Dementia - Parkinson’s Disease - Cardiovascular disease - Cancer – specify _____________ - COPD - Diabetes - Multiple Sclerosis - Epilepsy | | | - Hypertension - Osteoporosis - Arthritis - Depression - Asthma - Motor Neuron Disorder - Polymyositis - Other_______________________   ___________________________ | |
| **Dentition (own, edentulous, dentures)** | Own/Dentures/Edentulous | | | | |
| **Diet/Fluid consistency** | - NBM - PEG - JJ Tube - Other _________ | - Liquidized - Puree - Minced Moist - Soft Bite Sized - Easy Chew - Normal Diet | | | - Extremely Thick - Moderately Thick - Mildly Thick - Slightly Thick - Thin fluids |
| **MMSE score/date last assessment** | Score: | | Date: | | |
| **Independence in oral care** | Yes/no/maybe/unsure | | | | |
| **Living status** | Independent living/ACF (check terminology) | | | | |

1. **Safety considerations**

This study involves a new intervention with a vulnerable population. However, the intervention uses a device that has Therapeutic Goods Approval and the residents who will be using this device will be monitored by care staff during the intervention.

Potential Risks and Solutions for residents undertaking intervention

| **Potential Risks** | **Solutions** |
| --- | --- |
| Aspiration of saliva secondary to increased saliva production during device use | Aspiration of saliva occurs daily. The continued use of usual oral care during this study to ensure adequate oral hygiene should assist with reducing complications that may arise from aspiration of saliva  Monitoring by care staff to ensure participants have adequate salvia management/control  If coughing occurs through use of the device and increased saliva production, a referral will be made for speech pathology at Maroba to review swallowing function (and appropriateness for continuation in study) |
| Initial discomfort to gums/jaw with the action of chewing the device | The treatment intervention has been staged in time use of the device, increasing weekly to allow for adjustment to oral comfort. If oral discomfort is identified/reported, a review by the researcher and speech pathologist at Maroba Aged care will take place and recommendations will be made for continuation in the study.  Sizing and selection of the device has been considered for oral comfort and ease placing and removing the device from the oral cavity with an eternal tab/handle. |
| Infection control | Infection control procedures are outlined in the treatment manual and are consistent with Covid 19 safety and cleaning measures. Staff will be trained in cleaning protocols for the device (which are not aerosol generated) to reduce the risk of infection. Daily cleaning will be tracked with the completion of a daily check list by care staff at the ACF. |

The risks to the participants undertaking intervention is considered negligible to low risk. There is low foreseeable risk of harm or discomfort, and any foreseeable risk is no more than an inconvenience (National Statement on Ethical Conduct in Human Research, 2007).

There are no known risks for the care staff assisting with the intervention outside of usual oral care assistance.

Further considerations have been made below not specifically to risk, but rather in regard to storage and labelling, which may impact on infection control for the use of the device.

Device considerations and storage

| Potential Concern | Strategy |
| --- | --- |
| Device labelling/identification | Devices will be stored as per dentures in storage container specifically for the MyoMunchee, labelled with the participants identification number.  The MyoMunchee will be stored as per Maroba Aged Care protocol for storage and identification of usual oral hygiene products |
| Lost/misplaced device | In the event of the MyoMunchee being misplaced/lost a new replacement device will be provided |

1. **Data monitoring for safety**

Data monitoring will occur throughout the study for the safety of residents undergoing the intervention by way of:

- Pre and post intervention assessment
- During intervention with daily monitoring of completion of the trial intervention and completion of data sheets by care staff
- Weekly collection of data sheets to ensure no adverse events

Monitoring and reporting of adverse events (AE), serious adverse events (SAE) and unexpected events (UE) will be conducted as per Maroba Aged Care incident reporting system ‘ionMY’ (governance, risk management, compliance platform), and entered by care staff as per Maroba Aged Care incident reporting guidelines. Events will be reported to the principal investigator within 24 hours of the UE/AE/SAE and entered into the University of Newcastle adverse events form in Research Information Management System within 72 hours of being reported as per NHMRC (2016) guidelines.

All UE/AE/SAE will be:

- assessed by the principal investigator using the University of Newcastle risk assessment matrix and NHMRC (2016) guidelines of level of severity

Grading system for AE

| **Level of severity** | **Criteria of level of severity** |
| --- | --- |
| Mild | Requires minimal/no treatment and do not interfere with daily activities |
| Moderate | Results in low level inconvenience or concern. May cause some interference with function |
| Severe | Interrupts usual daily activity and may require other treatment |

- Reported to the sponsor and trial site by the principal investigator within 24 hours of the principal investigator becoming aware of any urgent safety concerns

Discontinuation of intervention for a participant may occur if:

- There is an AE or medical condition that occurs such that participation in the study would not be in the best interest of the participant
- The participant met an exclusion criterion (newly developed of not identified on consent) that precludes further study participation

Care staff who have concerns that a participant is no longer suitable to continue the intervention will be able to contact the principal investigator both during work hours and after hours via phone/email provided.

1. **Protocol Deviations**

Acknowledgment of the completion of twice daily intervention with the MyoMunchee will be confirmed by care staff via a checklist provided. The checklist will be collected by care staff at the end of each intervention week and replaced with a new intervention checklist with instructions for the intervention for that week.

Deviations from the study protocol such as the use of the device or adherence of the participant or the study site staff will be addressed by the principal investigator. The principal investigator will use continuous vigilance to identify and report deviations within 72 hours of identification of the protocol deviation.

1. **Unexpected or Serious Adverse Events**

The study procedures are not greater than minimal risk, and serious adverse events (SAE) are not expected.

- AE’s are those resulting in any untoward medical occurrence as a result of the intervention
- SAE’s are those resulting in death, life threatening AE’s, requiring hospitalization, or significant disruption of ability to conduct normal functions
- UE’s are those incidents/experiences that warrant consideration of change to the intervention protocol for the safety and welfare of the participant (National Health and Medical Research Council (2016).

The principal investigator will use continuous vigilance to identify and report adverse events within 72 hours of identification of the event to all approving relevant parties.

# DATA ANALYSIS

**a. Quantitative**

Statistical analyses will be performed using SPSS, version 22. The primary outcome will be reported as numbers and percentages.

Secondary outcome measures will be summarised using mean (SD) for normally distributed data, median (interquartile range) for non-normally distributed data and number (percent for categorical data).

Pre- to post-treatment comparisons will be performed using a paired t-test. Effect size and 95% confidence interval will be reported. The significance level will be set at p<0.05.

In addition, for each of the secondary outcome measures, linear regression will be used to measure changes in secondary variables while controlling for age, cognition, adherence, comorbidities, and ability to implement the MyoMunchee independently.

**b. Qualitative**

Qualitative content analysis as described by Graneheim and Lundman (2004) will be used to analyse participants' responses on both the resident survey and care staff post intervention questionnaire.

**c. De-identification**

All residents who are consented to the study where biographical data is collected will be assigned a resident ID number. The participants identity will not be identifiable to those outside of the research team.

# DATA HANDLING AND RECORD KEEPING

1. **Data Collection and Management Responsibilities**

The study will adhere to The Australian Code for the Responsible Conduct of Research (2018) as well as the University of Newcastle Data management policy (2017).

Data collection and management is the responsibility of the research team from the University of Newcastle and Maroba Aged Care, under the supervision of the Principal Investigator. The care staff are responsible for ensuring the accuracy, completeness, legibility, and timeliness of the data collection during the intervention.

The data and biographical information will be collected in paper form by an employee appointed by Maroba Aged Care. The data will be deidentified using a code and then provided to the research team. The data will be transferred by the research team while onsite at Maroba Aged Care to a University of Newcastle supported data storage platform (OneDrive/Teams) that is password protected and accessed for analysis by the research team.

1. **Study Records Retention**

The Principal Investigator will maintain all records pertaining to this study and will be retained as per the [State Records Act 1998](https://policies.newcastle.edu.au/directory-summary.php?legislation=26) and the University of Newcastle Data Management Policy (2017) for 5 years at the University of Newcastle.

Research data and primary materials will be retained for sufficient time to allow them to be referenced by other researchers and interested parties. In the event that results from research are challenged, all associated research data and materials must be retained until the matter is resolved.

Research data and primary materials will be disposed of in accordance with the relevant legislated minimum retention period of 5 years and with the consent of the research team as per the Australian Code for the Responsible Conduct of Research (2018).

# PUBLICATION & INTELLECTUAL PROPERTY

1. **Dissemination of results to participants**

Participants on signing the consent form for participation can elect to be notified of the results of the study, and the format this is received (electronic, hard copy of the results). This will be outline further in the Participant Information Sheet.

# ETHICAL CONSIDERATIONS

All eligible and consented participants will be informed of the potential risks and proposed benefits of the study procedures, the priority of the participants’ interests over those of science or of society.

The Myofunctional Device (MyoMunchee
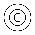
) has been approved by the Australian Government Department of Health (2019), Therapeutic Goods Administration as a medical device class 1 (low risk device), with intended purpose to exercise the jaw and surrounding muscles, as well as other conditions treated by oral motor therapy. The myofunctional device (MyoMunchee
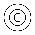
) is ISO 13485 certified by the Standard for Quality Management System for design and manufacturing of medical devices, and is made of non-toxic medical grade silicone.

1. **Vulnerable populations**

This study will involve vulnerable populations, including the elderly and those with chronic health conditions (including cognitive difficulties such as dementia). Informed consent will be obtained from residents/family members of residents if resident unable to provide consent/care staff to take part in this research. Residents/family members of residents/care staff will be required to read and understand the information (Participant Information Statement (PIS)) provided by the research team they need to make an informed decision about their voluntary participation. There will be allowances made for those who cannot read but would like to participate and the PIS can be read to them. Participants/family members of participants who consent to the intervention are permitted to change their mind and withdraw from participation at any time during the study.

1. **Confidentiality**

Confidentiality of all records is guaranteed and no information by which a person can be identified will be released and only anonymous data will be published. Participants will be assigned a pseudonym for the purposes of presentation/further dissemination of the research in order to protect their privacy and confidentiality. All files will be password protected and only accessible by the research team at the University of Newcastle.

1. **Ethical Review**

The study will be conducted in full conformance with principles of the National Statement on Ethical Conduct in Human Research (NHMRC, 2007), Australian Code for the Responsible Conduct of Research (2007) and within the laws and regulations Australia.

Ethical approval will be sought from the following:

- University of Newcastle Ethics committee
- Maroba Aged Care

# OUTCOMES AND SIGNIFICANCE

The outcomes from this study of feasibility and efficacy of test procedures for the use of a myofunctional device in improving in oral health and dysphagia in an aged care population, will further guide the design of a randomized control trial.

**REFERENCES**

1. Ajwani, S., Jayanti, S., Burkolter, N., Anderson, C., Bhole, S., Itaoui, R., & George, A. (2017). Integrated oral health care for stroke patients - a scoping review. *Journal of clinical nursing*, *26*(7-8), 891–901. <https://doi.org/10.1111/jocn.13520>
2. AHMAC (Australian Health Ministers Advisory Council) 2017. Aboriginal and Torres Strait Islander Health Performance Framework 2017 Report. Canberra: AHMAC.
3. Australian Institute of Health & Welfare (2021) Oral Health and Dental Care in Australia. <https://www.aihw.gov.au/reports/dental-oral-health/oral-health-and-dental-care-in-australia/contents/introduction>
4. Belafsky, P.C., Mouadeb, D.A., Rees, C.J., Pryor, J.C., Postma, G.N., Allen, J., & Leonard, R.J. (2008). Validity and reliability of the Eating Assessment Tool (EAT-10). Annuals Otolaryngology Rhinolaryngology & Laryngology. Dec;117(12):919-24. doi: 10.1177/000348940811701210. PMID: 19140539.
5. Bourke, K. (n.d.). 9th Asian Pacific Conference The Chewing Brush - Oral Physiotherapy - Lecture, 12. Retrieved 28^th^ June 2021 from <https://static1.squarespace.com/static/57ba3862893fc01e071991d8/t/5912949f2e69cf5fef74e9fb/1494389920781/The+Chewing+Brush.pdf>
6. Chalmers, J., Johnson, V., Tang, J.H., & Titler, M.G. (2004). Evidence-based protocol: oral hygiene care for functionally dependent and cognitively impaired older adults. *Journal Gerontology Nursing.* Nov;30(11):5-12
7. Council of Australian Governments Health Council (2019). Healthy mouths, healthy lives: Australia’s National Oral Health Plan 2015–2024. Performance monitoring report: baseline report 2017. Victoria: Dental Health Services Victoria.
8. Crary, M.A., Carnaby-Mann, G.D., & Groher, M.E. (2005). Initial psychometric assessment of a functional oral intake scale for dysphagia in stroke patients. *Arch Phys Med Rehabilitation,*86:1516-1520
9. Danckert, R., Ryan, A., Plummer, V., & Williams, C. (2016). Hospitalisation impacts on oral hygiene: an audit of oral hygiene in a metropolitan health service. *Scandinavian journal of caring sciences*, *30*(1), 129–134. https://doi.org/10.1111/scs.12230
10. Drapal, C. S. (2015). *Oral Care Practice Guidelines for the Care-Dependent Hospitalized Adult Outside of the Intensive Care Unit Setting*.
11. Eldridge, S.M., Chan, C.L., Campbell, M.J., Bond, C.M., Hopewell, S., & Thabane, L. (2010). CONSORT 2010 statement: extension to randomised pilot and feasibility trials  *BMJ*2016;  355 :i5239 doi:10.1136/bmj.i5239
12. Farrell, D. C., & Darcy, M. (2018, May/June). The history of myofunctional orthodontics: Part 1. The beginning & the separation. *Australian Dental Practice, special report, 74-76.*
13. Hägglund, P.,  Koistinen, S.,  Olai, L.,  Ståhlnacke, K.,  Wester, P.,  Levring Jäghagen, E.  (2019). Older people with swallowing dysfunction and poor oral health are at greater risk of early death. *Community Dent Oral Epidemiol*. 47: 494– 501. <https://doi.org/10.1111/cdoe.12491>
14. Hägglund, P., Hägg, M., Wester, P., Levring Jäghagen, E. (2019). Effects of oral neuromuscular training on swallowing dysfunction among older people in intermediate care—a cluster randomised, controlled trial, Age and Ageing, Volume 48, Issue 4, July 2019, Pages 533–540, <https://doi.org/10.1093/ageing/afz042>
15. Huang, S., Chiou, C., & Liu, H. (2017). Risk factors of aspiration pneumonia related to improper oral hygiene behavior in community dysphagia persons with nasogastric tube feeding. Journal of Dental Sciences. 12. 10.1016/j.jds.2017.06.001
16. Huang, Y.-S., Chuang, L.-C., Hervy-Auboiron, M., Paiva, T., Lin, C.-H., & Guilleminault, C. (2018). Neutral supporting mandibular advancement device with tongue bead for passive myofunctional therapy: A long term follow-up study. *Sleep Medicine*. <https://doi.org/10.1016/j.sleep.2018.09.013>
17. Huckabee, M.L., McIntosh, T., Fuller, L., Curry, M., Thomas, P., Walshe, M., McCague, E., Battel, I., Nogueira, D., Frank, U., van den Engel-Hoek, L., & Sella-Weiss, O. (2018). The Test of Masticating and Swallowing Solids (TOMASS): reliability, validity and international normative data. International Journal of Language and Communication Disorders. Jan;53(1):144-156. doi: 10.1111/1460-6984.12332. Epub 2017 Jul 5. PMID: 28677236.
18. Hughes, T.A., Wiles, C.M. (1996). Clinical measurement of swallowing in health and in neurogenic dysphagia. *QJM.*89:109–16.
19. In, J. (2017). Introduction of a pilot study. *Korean journal of anesthesiology*, *70*(6), 601–605. <https://doi.org/10.4097/kjae.2017.70.6.601>
20. Korbmacher, H. M., Schwan, M., Berndsen, S., Bull, J., & Kahl-Nieke, B. (2004). Evaluation of a new concept of myofunctional therapy in children. *The International Journal of Orofacial Myology: Official Publication of the International Association of Orofacial Myology*, *30*, 39–52.
21. Langmore, S. E., Terpenning, M. S., Schork, A., Chen, Y., Murray, J. T., Lopatin, D., & Loesche, W. J. (1998). Predictors of Aspiration Pneumonia: How Important Is Dysphagia? *Dysphagia*, *13*(2), 69–81. <https://doi.org/10.1007/PL00009559>
22. Luong, E., Drysdale, P., Campbell, E., deClifford, J., Lam, S. H., Coutsouvelis, J, & Danckert, R. (2018). Implementation of standardised oral care treatment and referral guidelines for older sub-acute patients: A multidisciplinary approach. *Journal of Pharmacy Practice and Research*, *48*(1), 10–17. <https://doi.org/10.1002/jppr.1311>
23. Manabe T, Teramoto S, Tamiya N, Okochi J, Hizawa N (2015) Risk Factors for Aspiration Pneumonia in Older Adults. PLOS ONE 10(10): e0140060.<https://doi.org/10.1371/journal.pone.0140060>
24. Mann, G. (2002). *MASA, The Mann Assessment of Swallowing Ability*. Vol. 1 New York, NY: Cengage Learning.
25. Moeller, J. L., Paskay, L. C., & Gelb, M. L. (2014). Myofunctional Therapy. A Novel Treatment of Pediatric Sleep-Disordered Breathing. *Sleep Medicine Clinics*, *9*, 235–243. <https://doi.org/10.1016/j.jsmc.2014.03.002>
26. Molloy, W., & Clarnette, R., (1999). Standardized Mini-Mental State Examination, A User’s Guide.Troy, Ont. : New Grange Press
27. Murray, J., & Scholten, I. (2018). An oral hygiene protocol improves oral health for patients in inpatient stroke rehabilitation. *Gerodontology, 35*, 18–24.
28. National Health and Medical Research Council (2016). Guidance: Safety monitoring and reporting in clinical trials involving therapeutic goods. Canberra: National Health and Medical Research Council.
29. Ray, J. (2006). Orofacial myofunctional deficits in elderly individuals. International Journal of Orofacial Myology, 32(1), 22-31.DOI: https://doi.org/10.52010/ijom.2006.32.1.2
30. Santoro, P., e Silva, I. L., Cardoso, F., Dias, E., Jr, & Beresford, H. (2011). Evaluation of the effectiveness of a phonoaudiology program for the rehabilitation of dysphagia in the elderly. *Archives of gerontology and geriatrics*, *53*(1), e61–e66. https://doi.org/10.1016/j.archger.2010.10.026
31. Shivayogi P. (2013). Vulnerable population and methods for their safeguard. *Perspectives in clinical research*, *4*(1), 53–57. <https://doi.org/10.4103/2229-3485.106389>
32. Shortland, H. L., Hewat, S., Vertigan, A., & Webb, G. (2021). Orofacial Myofunctional Therapy and Myofunctional Devices Used in Speech Pathology Treatment: A Systematic Quantitative Review of the Literature. *American journal of speech-language pathology*, *30*(1), 301–317. https://doi.org/10.1044/2020_AJSLP-20-00245
33. Slade GD, Spencer AJ, Roberts-Thomson KF (Editors) 2007. Australia’s dental generations: the National Survey of Adult Oral Health 2004–06. *Dental Statistics and Research series no. 34.* AIHW cat. no. DEN 165. Canberra: AIHW.
34. Thabane, L., Lancaster, G.A., (2019) Guide to the reporting of protocols of pilot and feasibility trials. *Pilot Feasibility Stud*ies. <https://doi.org/10.1186/s40814-019-0423-8>
35. Therapeutic Goods Administration (2019). Australian Government Department of Health and Therapeutic Goods. <https://www.ebs.tga.gov.au/servlet/xmlmillr6?dbid=ebs/PublicHTML/pdfStore.nsf&docid=C70844B99008C793CA25844F00422D18&agid=(PrintDetailsPublic)&actionid=1>
36. Wang, T. F., Huang, C. M., Chou, C., & Yu, S. (2015). Effect of oral health education programs for caregivers on oral hygiene of the elderly: A systemic review and meta-analysis. *International journal of nursing studies*, *52*(6), 1090–1096. https://doi.org/10.1016/j.ijnurstu.2015.01.015
37. Wishney, M., Darendeliler, M. A., & Dalci, O. (2019). Myofunctional therapy and prefabricated functional appliances: An overview of the history and evidence. *Australian Dental Journal*. <https://doi.org/10.1111/adj.12690>
