# Supplementary figures and images for: Myofunctional device use in oral care and swallowing: a protocol for a feasibility study in an aged care population
Source: Pilot Feasibility Stud. 2022 Aug 19;8:187. doi: 10.1186/s40814-022-01148-3 (PMC9388991; doi:10.1186/s40814-022-01148-3)

Appendix B


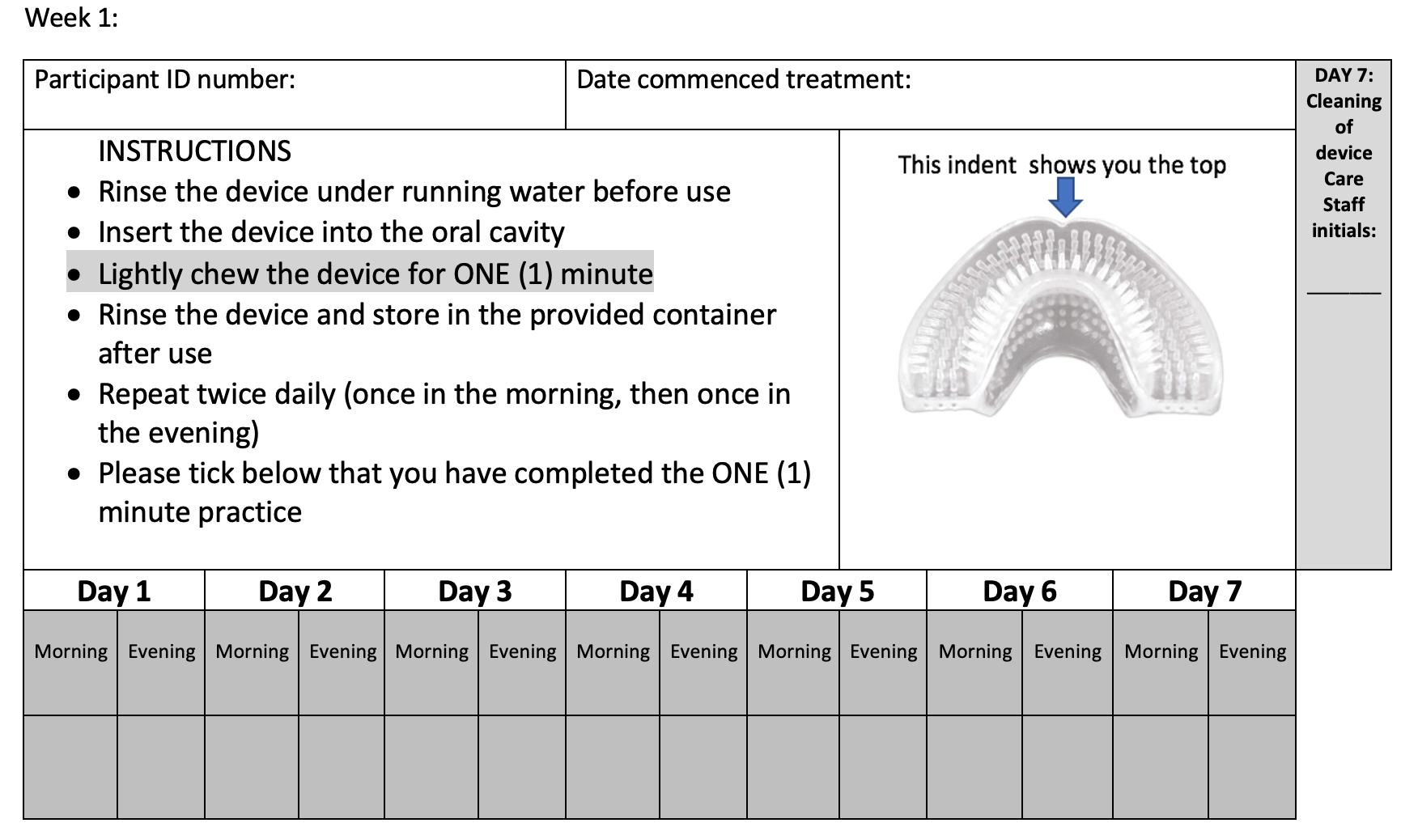

Supplement: Supplementary file 2 — Additional file 2. Protocol. Week 1. [file 40814_2022_1148_MOESM2_ESM.docx]
